# Tofacitinib for child-onset systemic lupus erythematosus

**DOI:** 10.3389/fimmu.2024.1457821

**Published:** 2024-10-08

**Authors:** Ling Hou, Peng Zhou, Chengguang Zhao, Xiuli Wang, Yue Du

**Affiliations:** Department of Pediatrics, Shengjing Hospital of China Medical University, Shenyang, China

**Keywords:** systemic lupus erythematosus (SLE), child, tofacitinib, jak-stat, DMARD

## Abstract

**Background:**

Systemic lupus erythematosus (SLE) is a chronic autoimmune disease that can cause diverse clinical manifestations in multiple organ systems. Child-onset SLE (cSLE) is associated with significantly higher morbidity and mortality than adult-onset SLE. The traditional treatments for SLE (glucocorticoids, antimalarials, conventional and biological disease-modifying antirheumatic drugs) often have significant adverse effects and may not fully control disease activity. Tofacitinib is an oral Janus kinase (JAK) inhibitor that inhibits the JAK-STAT pathway and has the potential to reduce SLE severity.

**Methods:**

cSLE patients who received tofacitinib and had at least one follow-up visit were retrospectively examined. Case histories, laboratory test results, and treatment regimens were analyzed at disease onset, initiation of tofacitinib treatment, and 1, 3, 6, 9, 12, 18, and 24 months after starting tofacitinib.

**Results:**

We examined 9 patients with refractory cSLE. All patients were female and the average age at diagnosis was 10.67 years. At initiation of tofacitinib, the average age was 13.28 years and the average disease duration was 2.62 years. Four patients experienced alleviation of symptoms and reduced their daily prednisone dosages; one of them also discontinued cyclosporine A and two of them also discontinued belimumab. The other 5 patients experienced no apparent benefit.

**Conclusion:**

Tofacitinib may provide clinical benefits for some patients with refractory cSLE, and can also allow reduction in the glucocorticoid dosage. Tofacitinib has the potential as an adjunctive treatment for some patients with cSLE.

## Introduction

Systemic lupus erythematosus (SLE) is a chronic autoimmune disease with clinical manifestations of varying severity in multiple organs and organ systems. Although adult-onset SLE is more common, child-onset SLE (cSLE) is typically more severe, and is associated with a higher mortality rate, more frequent organ involvement, higher disease activity, and the potential need for long-term high-dose immunosuppressive therapy (1, 2). The management of cSLE is particularly challenging because children generally have poor tolerance of the medications typically used to control adult SLE (1, 3). The current treatment options for cSLE include glucocorticoids, antimalarials, and conventional and biological disease modifying drugs (DMARDs), but these can have significant adverse effects and may not fully control disease activity (4, 5).

Tofacitinib is an oral Janus kinase (JAK) inhibitor that has high selectivity for JAK1 and JAK3, and recent studies reported it had potential as a treatment for cSLE (4, 6–10). Cytokines such as type I interferon (IFN), interleukin 12 (IL-12), and IL-23 (which connect the innate and adaptive immune systems), and IL-21 (which activates interactions of T-cells and B-cells) contribute to the characteristic inflammation of SLE. Activation of these cytokines requires increased JAK-mediated signaling: type I IFN activates JAK1/tyrosine kinase 2 (TYK2); IL-12 and IL-23 activate JAK2/TYK2; and IL- 21 activates JAK1/JAK3. Because JAK inhibitors block the signaling of multiple cytokines, they are a promising class of drugs for the treatment of SLE, which has heterogeneous immune manifestations (4, 8, 11–13). The tofacitinib-mediated inhibition of the JAK-STAT pathway therefore appears to be theoretically well-suited for treatment of SLE. In fact, tofacitinib has demonstrated good efficacy as a treatment for several other autoimmune diseases, including adult rheumatoid arthritis (14–16), and recent studies have examined its possible efficacy and safety as a treatment for SLE (8, 9, 17, 18).

This study retrospectively collected clinical data of patients with refractory cSLE who received tofacitinib at our hospital, and analyzed multiple clinical observations to assess its potential efficacy and safety as a treatment for cSLE. Given the difficulties that cSLE patients often face, and their failure to achieve long-term disease control without adverse effects, it is crucial to identify new treatments. This study aimed to assess the potential for expanding the use of JAK inhibitors by examining the effect of tofacitinib on refractory cSLE.

## Methods

### Patients

All included cSLE patients were treated at the Pediatric Nephrology and Rheumatology Department of Shengjing Hospital, China Medical University, between January 2013 and December 2023, and received tofacitinib treatment with at least one follow-up visit. Concurrent use of other medications (glucocorticoids, antimalarials, conventional and biological DMARDs, etc.) was allowed. The diagnosis of SLE was based on the 1997 American College of Rheumatology (ACR) criteria (19), the 2012 Systemic Lupus International Collaborating Clinics (SLICC) revised criteria (20), or the 2019 European League Against Rheumatism/American College of Rheumatology (EULAR/ACR) criteria (21).

### Data collection

Patient data were collected retrospectively, and included general information (gender, age, and disease duration), clinical symptoms (fever, rash, arthritis, oral ulcers, photosensitivity, hair loss, serositis, etc.), and laboratory test results (white blood cell count, platelet count, serum complement levels, anti-dsDNA antibody status, urine protein quantification, urine sediment, red blood cell count, SLE Disease Activity Index-2000 score [SLEDAI-2K, range: 0–105], and SLE Damage Index [SDI, range: 0–47]). These data were collected at disease onset, at the start of tofacitinib treatment, and at 1, 3, 6, 9, 12, 18, and 24 months after initiation of tofacitinib. Records of previous treatments, including use of glucocorticoids, antimalarials, and conventional and biological DMARDs, were also analyzed.

### Evaluation of treatment efficacy and adverse reactions

The efficacy of tofacitinib in each patient was classified into one of the following four groups:

Complete Remission: No disease activity without the use of glucocorticoids, but with the use of antimalarials allowed.

Clinical Remission: Clinically quiescent but serologically active disease, with high levels of serological markers (anti-dsDNA positivity and/or hypocomplementemia), with prednisone dosage below 5 mg/day.

Alleviated: Some resolution of clinical symptoms and improvement of laboratory test results.

Unalleviated: No resolution or further deterioration of clinical symptoms and laboratory test results.

At each follow-up visit after the initiation of tofacitinib treatment, all adverse events were recorded in detail.

### Ethics

Patients and their guardians were provided with thorough information about tofacitinib, and were told it is a novel oral protein tyrosine kinase inhibitor that was approved by the U.S. FDA for treatment of polyarticular juvenile idiopathic arthritis in children aged 2 years and older, but that it is not yet approved for treatment of cSLE in China. Written informed consent was obtained from all patients and their guardians before treatment, and these records were kept in the medical files. Because this clinical study is descriptive and retrospective, it did not require approval from the Ethics Committee, according to Chinese regulations. After providing informed consent, all patients and their guardians consented to the use of the data for research purposes.

## Results

### Clinical characteristics of patients

We included 9 children with refractory cSLE in this study (**Table 1**). All the children were females and the average age at diagnosis of cSLE was 10.67 years (range: 7–13 years). The average age at the start of tofacitinib treatment was 13.28 years (range: 11.25–15.75 years) and the average disease duration at the start of tofacitinib treatment was 2.62 years (range: 0.03–8.75 years). The average SLEDAI-2K score at disease onset was 11.11 (range: 7–16); 6 patients had moderate disease activity (SLEDAI-2K score: 7–12) and 3 patients had severe disease activity (SLEDAI-2K: >12). Cases 3, 7, and 8 had SDI scores of 1. Three patients also had concurrent lupus nephritis (LN), 1 each with class IV, class I, and class IV+V. All patients used several medications before starting tofacitinib (range: 2–6), including glucocorticoids, hydroxychloroquine (HCQ), conventional DMARDs (mycophenolate mofetil [MMF], cyclophosphamide [CTX], tacrolimus, cyclosporine A [Cys A], leflunomide [LEF]), and a biological DMARD (belimumab). All patients were given tofacitinib because of difficulty in reducing the glucocorticoid dose or glucocorticoid-induced growth retardation (7 patients), disease relapse (1 patient), or persistent hair loss (1 patient).

**Table 1:** Characteristics of patients before tofacitinib and at initiation of tofacitinib.

Patient	Sex	Age at diagnosis (years)	SLEDAI-2K at diagnosis	Prior treatments	Main reason for TOF initiation	Disease duration at TOF initiation (years)	Age at TOF initiation (years)	Main clinical manifestations at TOF initiation
1	F	10.00	8	GC, CTX, MMF HCQ, Cys A belimumab	Growth retardation from GC	3.50	13.50	Arthralgia, fever, thrombocytopenia
2	F	11.00	7	GC, HCQ, MMF, belimumab	Difficulty tapering GC	2.50	13.50	Rash, fever, oral ulcer
3	F	11.00	16	GC, HCQ, belimumab, tacrolimus, CTX, LEF	Difficulty tapering GC	0.92	11.92	Rash, fever, arthralgia, oral ulcer, hair loss, LN (IV)
4	F	11.00	10	GC, HCQ, tacrolimus, belimumab	Disease flare	0.75	11.75	Arthritis, fever, rash
5	F	13.00	13	GC, tacrolimus, HCQ, belimumab	Difficulty tapering GC	0.03	13.03	Rash, fever, thrombocytopenia, LN (I)
6	F	10.00	8	GC, HCQ, MMF	Hair loss, no remission	5.42	15.42	Rash, hair loss
7	F	13.00	12	GC, MMF	Difficulty tapering GC	0.42	13.42	Thrombocytopenia, fever
8	F	10.00	14	GC, HCQ, CTX, MMF, belimumab	Growth retardation from GC	1.25	11.25	Rash, fever, LN (IV+V)
9	F	7.00	12	GC, HCQ, CTX, MMF, belimumab	Difficulty tapering GC	8.75	15.75	Thrombocytopenia, rash

TOF, tofacitinib; F, Female; GC, glucocorticoid; CTX, cyclophosphamide; MMF, mycophenolate mofetil; HCQ, hydroxychloroquine; Cys A, cyclosporine A; LEF, leflunomide; LN, lupus nephritis; SLE, systemic lupus erythematosus; SLEDAI-2K, SLE Disease Activity Index-2000.

### Efficacy and adverse reactions to tofacitinib

**Figure 1:**
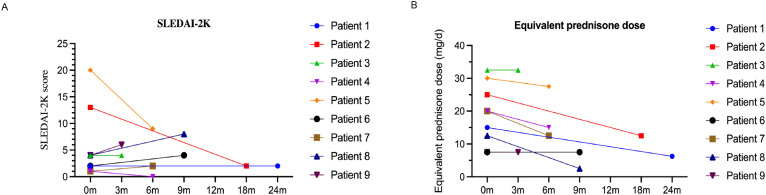
Changes in SLEDAI-2K score **(A)** and the dosage of prednisone **(B)** during follow-up.

We analyzed the dosing, efficacy, and safety of tofacitinib in all 9 patients, each of whom had at least one follow-up visit between January 2013 and December 2023 (**Table 2**). Specific information for each of the nine cases is given below. The median SLEDAI-2K score of the 9 patients at tofacitinib initiation was 4.00 (interquartile range: [IQR]: 1.50, 8.50). At the last follow-up, the median SLEDAI-2K score was 2.00 (IQR: 1.00, 6.00) (**Figure 1A**), with no significant difference. The SDI score was 0 in all cases at tofacitinib initiation; case 5 had an SDI score of 1 at the last follow-up. Among all 9 patients, 4 cases (1, 2, 4, and 7) experienced disease alleviation and continued this therapy, and the other 5 cases experienced no disease alleviation and discontinued this therapy. The 4 patients who achieved alleviation all reduced their dosages of prednisone (case 1: 15 mg/day to 6.25 mg/day, case 2: 25 mg/day to 12.5 mg/day, case 4: 20 mg/day to 15 mg/day, case 7: 20 mg/day to 12.5 mg/day; **Figure 1B**). In addition, case 1 discontinued CysA, and cases 2 and 4 discontinued belimumab. The 5 cases who discontinued tofacitinib received other immunosuppressants (cyclophosphamide, tacrolimus, or others) to control disease activity and reduce corticosteroid usage (**Table 2**), as described below.

**Table 2:** Treatment regimens and outcomes of cSLE patients who received tofacitinib (5 mg bid).

Patient	Concomitant treatments at TOF initiation	SLEDAI-2K at TOF initiation	TOF duration (months)	TOF use at last follow-up	Response as of last recorded treatment	Additional treatments at last follow-up	SLEDAI-2K at last follow-up	Abnormal laboratory findings at last follow-up	Adverse effects as of last recorded treatment
1	GC (15 mg qd), HCQ (100 mg bid), Cys A (morning 50 mg, evening 25 mg), belimumab (480 mg q4w)	2	24	Ongoing	Alleviated	GC (6.25 mg qd), HCQ (100 mg bid), tofacitinib (5 mg bid), telitacicept (80 mg qw)	2	anti-dsDNA positive	None
2	GC (25 mg qd), HCQ (150 mg bid), MMF (0.5 g bid), belimumab (480 mg q4w)	13	18	Ongoing	Alleviated	GC (12.5 mg qd), HCQ (150 mg bid), MMF (0.5 g bid), tofacitinib (5 mg bid)	2	anti-dsDNA positive	None
3	GC (32.5 mg qd), HCQ (100mg bid), LEF (20 mg qd), MMF (morning: 0.5g, evening: 0.25 g)	4	12	Discontinued	Unalleviated	GC (5 mg qd), HCQ (100 mg bid), MMF (morning: 0.5 g, evening: 0.25 g)	4	None	None
4	GC (20 mg qd), HCQ (100 mg bid, tacrolimus (1 mg bid), belimumab (360 mg q4w)	1	6	Ongoing	Alleviated	GC (15 mg qd), HCQ (100 mg bid), tacrolimus (1 mg bid), tofacitinib (5 mg bid)	0	None	None
5	GC (30 mg qd), HCQ (100 mg bid), Tacrolimus (1 mg bid), belimumab (480 mg q4w)	20	6	Discontinued	Unalleviated	GC (27.5 mg qd), HCQ (100 mg bid), LEF (20 mg qd), belimumab (480 mg q4w)	9	proteinuria, decreased C3, anti-dsDNA positive, decreased WBC	upper respiratory tract infection
6	GC (7.5 mg qd), HCQ (100 mg bid), MMF (0.5 g bid)	2	9	Discontinued	Unalleviated	GC (7.5 mg qd), HCQ (100 mg bid), MMF (0.5 g bid)	4	decreased C3, anti-dsDNA positive	None
7	GC (20 mg qd), MMF (morning: 0.75 g, evening: 0.5 g)	1	6	Ongoing	Alleviated	GC (12.5 mg qd), MMF (morning: 0.75 g, evening: 0.5g), tofacitinib (5 mg bid)	2	anti-dsDNA positive	None
8	GC (12.5 mg qd), HCQ (morning: 100mg, evening: 50mg), CTX (350 mg q4w), MMF (morning: 0.5 g, evening: 0.25 g), belimumab (360 mg q4w)	4	9	Discontinued	Unalleviated	GC (2.5 mg qd), HCQ (morning: 100mg, evening: 50mg), MMF (0.5 g bid), tacrolimus (1 mg bid)	8	proteinuria, decreased C3, anti-dsDNA positive	None
9	GC (15 mg qod), HCQ (100 mg bid), MMF (0.5 g bid), belimumab (600 mg q4w)	4	24	Discontinued	Unalleviated	GC (5 mg qod), HCQ (100 mg bid), MMF (0.5 g bid), tacrolimus (1 mg bid)	6	anti-dsDNA positive	None

TOF, tofacitinib; GC, glucocorticoid; CTX, cyclophosphamide; MMF, mycophenolate mofetil; HCQ, hydroxychloroquine; Cys A, cyclosporine A; LEF, leflunomide; LN, lupus nephritis; SLE, systemic lupus erythematosus; WBC, white blood cells; SLEDAI-2K, SLE Disease Activity Index-2000.

During the period of treatment with tofacitinib, 8 patients demonstrated good tolerance, based on follow-up periods of 6 to 24 months. However, case 5 experienced an upper respiratory tract infection (nasal congestion, mild cough).

#### Case 1

Disease duration: 3.5 years.

Prior treatment: prednisone (15 mg qd), HCQ (100 mg bid), Cys A (morning: 50 mg, evening: 25 mg), belimumab (480 mg q4w IV).

Reason for tofacitinib addition: Growth retardation due to prolonged glucocorticoid use.

Tofacitinib dosage: 5 mg bid.

Follow-up: 24 months, Cys A was discontinued, prednisone dosage was reduced (6.25 mg qd), belimumab changed to telitacicept (80 mg qw SQ), height increased by 5 cm.

SLEDAI-2K score: 2 (anti-dsDNA positivity).

Clinical outcome: Alleviated, tofacitinib continued, no adverse reactions.

#### Case 2

Disease duration: 2.5 years.

Prior treatment: prednisone (25 mg qd), HCQ (150 mg bid), MMF (0.5 g bid), belimumab (480 mg q4w IV).

Reason for tofacitinib addition: Difficulty reducing prednisone dose.

Tofacitinib dosage: 5 mg bid.

Follow-up: 18 months, prednisone dosage reduced (12.5 mg qd), belimumab discontinued.

SLEDAI-2K score: 2 (anti-dsDNA positivity).

Clinical outcome: Alleviated, tofacitinib continued, no adverse reactions.

#### Case 3

Disease duration: 0.92 years.

Complication: lupus nephritis class IV.

Prior treatment: prednisone (32.5 mg qd), HCQ (100 mg bid), LEF (20 mg qd), MMF (morning: 0.5 g, evening: 0.25 g).

Reason for tofacitinib addition: Difficulty reducing prednisone dose.

Tofacitinib dosage: 5 mg bid.

Follow-up: 3 months, prednisone could not be reduced, standard CTX pulse therapy (350 mg q4w) initiated.

SLEDAI-2K score: 4.

Follow-up at 12 months: prednisone dosage reduced (5 mg qd), LEF discontinued.

SLEDAI-2K score: 0.

Clinical outcome: Unalleviated, tofacitinib discontinued (6 months), no adverse reactions.

#### Case 4

Disease duration: 0.75 years.

Prior treatment: prednisone (20 mg qd), HCQ (100 mg bid), tacrolimus (1 mg bid), belimumab (360 mg q4w IV).

Reason for tofacitinib addition: Recurring fever and rash.

Tofacitinib dosage: 5 mg bid.

Follow-up: 6 months, prednisone dosage reduced (15 mg qd), belimumab discontinued.

SLEDAI-2K score: 0.

Clinical outcome: Alleviated, tofacitinib continued, no adverse reactions.

#### Case 5

Disease duration: 0.03 years.

Complication: lupus nephritis class I.

Prior treatment: prednisone (30 mg qd), HCQ (100 mg bid), tacrolimus (1 mg bid), belimumab (480 mg q4w IV).

Reason for tofacitinib addition: Difficulty reducing prednisone dose.

Tofacitinib dosage: 5 mg bid.

Follow-up: 6 months, prednisone dosage reduced (27.5 mg qd), added LEF (20 mg qd).

SLEDAI-2K score: 9 (moderate disease activity).

Clinical outcome: Unalleviated, tofacitinib discontinued, an upper respiratory tract infection occurred, and resolved with symptomatic treatment.

#### Case 6

Disease duration: 5.42 years.

Prior treatment: prednisone (7.5 mg qd), HCQ (100 mg bid), MMF (0.5 g bid).

Reason for tofacitinib addition: persistent hair loss, no remission.

Tofacitinib dosage: 5 mg bid.

Follow-up: 9 months, no change in hair loss.

SLEDAI-2K score: 4 (anti-dsDNA positivity, low serum complement C3).

Clinical outcome: Unalleviated, tofacitinib discontinued (9 months), no adverse reactions.

#### Case 7

Disease duration: 0.42 years.

Prior treatment: prednisone (20 mg qd), MMF (morning: 0.75 g, evening: 0.5 g), no HCQ due to visual field loss.

Reason for tofacitinib addition: difficulty reducing prednisone dose.

Tofacitinib dosage: 5 mg bid.

Follow-up: 6 months, prednisone dosage reduced (12.5 mg qd).

SLEDAI-2K score: 2 (anti-dsDNA positivity).

Clinical outcome: Alleviated, tofacitinib continued, no adverse reactions.

#### Case 8

Disease duration: 1.25 years.

Complication: lupus nephritis class IV+V.

Prior treatment: prednisone (12.5 mg qd), HCQ (morning: 100 mg, evening: 50 mg), CTX (350 mg q4w), MMF (morning: 0.5 g, evening: 0.25 g), belimumab (360 mg q4w IV).

Reason for tofacitinib addition: growth retardation due to prolonged glucocorticoid use.

Tofacitinib dosage: 5 mg bid.

Follow-up: 9 months, prednisone dosage reduced (2.5 mg qd), CTX and belimumab discontinued, MMF dosage adjusted (0.5 g bid), added tacrolimus (1 mg bid), height increased by 3 cm.

SLEDAI-2K score: 8 (proteinuria, decreased C3, anti-dsDNA positive).

Clinical outcome: Unalleviated, tofacitinib discontinued, no adverse reactions.

#### Case 9

Disease duration: 8.75 years.

Prior treatment: prednisone (15 mg qod), HCQ (100 mg bid), MMF (0.5 g bid), belimumab (600 mg q4w IV).

Reason for tofacitinib addition: difficulty reducing prednisone dose.

Tofacitinib dosage: 5 mg bid.

Follow-up: 3 months, no reduction in prednisone dosage, disease activity observed, SLEDAI-2K score: 6 (decreased C3, anti-dsDNA positive, fever, decreased WBC), added tacrolimus (1 mg bid).

Follow-up at 24 months: prednisone dosage reduced (5 mg qod).

SLEDAI-2K score: 2 (anti-dsDNA positivity).

Clinical outcome: Unalleviated, tofacitinib discontinued (3 months), no adverse reactions during follow-up.

## Discussion

SLE is a systemic autoimmune inflammatory disease of unknown etiology that has heterogeneous clinical manifestations, an uncertain clinical course, and highly variable patient prognosis. The goal of treatment is to achieve disease remission or lower disease activity and to improve patient quality-of-life (8, 13). Unlike other autoimmune diseases, there are no well-established biological DMARD therapies for SLE, although the current treatments include belimumab (22, 23), telitacicept (24, 25), and anti-CD20 monoclonal antibodies (26). However, the clinical outcomes from these molecular targeted therapies are still unsatisfactory, possibly because the highly heterogeneous immunological abnormalities in SLE patients mean that multiple cells and molecules must be targeted. Thus, a drug that has a single molecular target may not provide consistent efficacy in a population of these patients (8). This indicates the need to identify additional treatments for SLE or a treatment that can target multiple molecules or cells.

cSLE can affect organs and organ systems simultaneously or sequentially, and these patients may experience unpredictable flares, high morbidity, and high mortality (27). Antimalarials and glucocorticoids are still the most commonly used drugs for treating cSLE. MMF or intravenous CTX is often recommended as induction therapy for class III and class IV LN, and calcineurin inhibitors (Cys A, tacrolimus, and voclosporin) are also good options for treatment of cSLE patients with LN. Rituximab (a biological DMARD that targets CD20 on B cells) can be combined with another DMARD for refractory LN, and the U.S. FDA recently approved belimumab (a biological DMARD that targets B-cell activating factor) for the treatment of cSLE in children who are more than 5-years-old (27). Phase II randomized controlled trials in adult SLE patients showed that anti-IFN therapy (sifalimumab and anifrolumab) (27) and some JAK inhibitors (8, 9, 17, 18) provided beneficial effects. These recent findings led us to examine the JAK inhibitor tofacitinib as a treatment option for patients with refractory cSLE.

To our best knowledge, the present retrospective clinical cohort study of 9 female patients with refractory cSLE is the largest study to examine tofacitinib as a treatment for cSLE. Our results showed that adding oral tofacitinib to a treatment regimen led to disease alleviation in 4 patients, all of whom also reduced their daily prednisone dosages; 1 of these patients also discontinued cyclosporine A and 2 other patients also discontinued belimumab. As of the last follow-up, all four of these patients had low disease activity and continued using tofacitinib. However, the addition of tofacitinib provided no significant benefit for the other 5 patients. Nonetheless, because some cSLE patients appear to benefit from tofacitinib treatment, we suggest that this drug should be considered when other multi-drug treatments are ineffective.

Similar to our findings, other recent studies also reported therapeutic benefits of JAK inhibitors when given to patients with SLE. For example, Pin et al. (10) studied the effect of different JAK inhibitors in 7 patients with early-onset pediatric inflammatory disorders, including a 9-year-old girl who experienced resolution of most symptoms after standard combined therapy, but who had unresolved headache, fatigue, and irritability. After adding baricitinib and gradually reducing the glucocorticoid dose, this girl’s mood improved significantly and her erythrocyte sedimentation rate returned to normal. Another study of adults with SLE found that tofacitinib led to decreased arthritis and rash, and allowed use of a lower steroid dose (28). A recent study used the Cutaneous Lupus Erythematosus Disease Area and Severity Index (CLASI) score to assess skin manifestations in adults with mild-to-moderate SLE who received tofacitinib, and reported that 3 patients with refractory cutaneous lesions experienced improved CLASI scores (29). A case report confirmed the beneficial effect of tofacitinib for refractory alopecia in a patient with SLE (30). A recent study described a patient with periorbital discoid lupus erythematosus who experienced significant improvement after treatment with a 2% tofacitinib ointment (18).

Three clinical trials have examined the effect of tofacitinib on SLE. A phase Ib trial (NCT02535689) that began in 2015 evaluated the safety of tofacitinib in SLE patients (8). This study stratified patients with mild-to-moderate disease activity based on *STAT4* risk allele status, and compared the safety and tolerability of tofacitinib (5 mg bid) plus standard treatment with standard treatment plus placebo. Most adverse events in the tofacitinib group were mild-to-moderate upper respiratory tract infections that resolved spontaneously or after oral antibiotic treatment, and there were no reports of herpes zoster reactivation or any venous thromboembolism events (9). Two other ongoing phase I/II trials are examining the effects of oral tofacitinib on discoid lupus, cutaneous lupus, and SLE with moderate-to-severe skin symptoms (NCT03159936, NCT03288324) (8).

There is evidence that oral tofacitinib is well-tolerated. In fact, only one of our patients experienced a mild upper respiratory tract infection, and there were no other adverse reactions, consistent with previous reports (8). The present study was a single-center retrospective study with a small sample size that focused on refractory cSLE and showed that tofacitinib appeared to benefit some patients. However, larger-scale clinical trials are needed to validate these findings and to examine the benefit of introducing tofacitinib at different stages of cSLE treatment. Randomized controlled trials are also required to more comprehensively assess its efficacy and safety.

In conclusion, our study of the treatment of patients with refractory cSLE using a combination of oral tofacitinib and other drugs showed that some patients experienced improvements and were able to reduce their glucocorticoid dosages, and that this drug was well-tolerated with a low incidence of adverse reactions. However, due to our small sample size and the retrospective design of this study, larger and more thorough prospective studies are needed to verify these preliminary results and also to evaluate the long-term effects and safety of tofacitinib when used as a treatment for cSLE.

## Data Availability

The raw data supporting the conclusions of this article will be made available by the authors, without undue reservation.
